# Differentiating Primary Pancreatic Lymphoma Versus Primary Splenic Lymphoma: A Case Report

**DOI:** 10.1089/pancan.2020.0019

**Published:** 2021-03-29

**Authors:** Robert A. Ries, Christina L. Jacovides, Jennifer Rashti, Jerald Z. Gong, Charles J. Yeo

**Affiliations:** ^1^ Department of Surgery, Thomas Jefferson University Hospital, Philadelphia, Pennsylvania, USA.; ^2^ Division of Trauma, Surgical Critical Care and Emergency Surgery, Penn Presbyterian Medical Center, Philadelphia, Pennsylvania, USA.; ^3^ Department of Pathology, Anatomy and Cell Biology, Thomas Jefferson University Hospital, Philadelphia, Pennsylvania, USA.

**Keywords:** primary splenic lymphoma, primary pancreatic lymphoma, left upper quadrant mass

## Abstract

**Background:** Both primary pancreatic lymphoma (PPL) and primary splenic lymphoma (PSL) represent rare entities. PPL typically arises in the head of the pancreas but may arise in other locations also. PSL usually presents with nonspecific symptoms, including left upper quadrant pain, weight loss, and fever. This report describes a patient with a large left upper quadrant mass, which initially was believed to be a primary pancreatic mass, but which on final pathology appeared to be consistent with a PSL.

**Presentation:** The patient is a 64-year-old woman who initially presented with symptoms of left upper quadrant abdominal pain and distension; she subsequently was found to have an 18 cm heterogeneous mass arising from the pancreatic tail. She underwent a distal pancreatectomy with splenectomy. Final pathology confirmed a diffuse large B cell lymphoma arising from the splenic parenchyma.

**Conclusions**: Both PPL and PSL are rare causes of left upper quadrant masses. In this case, we describe a large lymphoma that appeared to arise from the tail of the pancreas, but on final pathology was found to be splenic in origin. Differentiating these two clinical entities is important for prognostication and treatment. A multimodal approach with surgical resection followed by chemotherapy is preferred.

## Presentation of Case

**FIG. 1.**
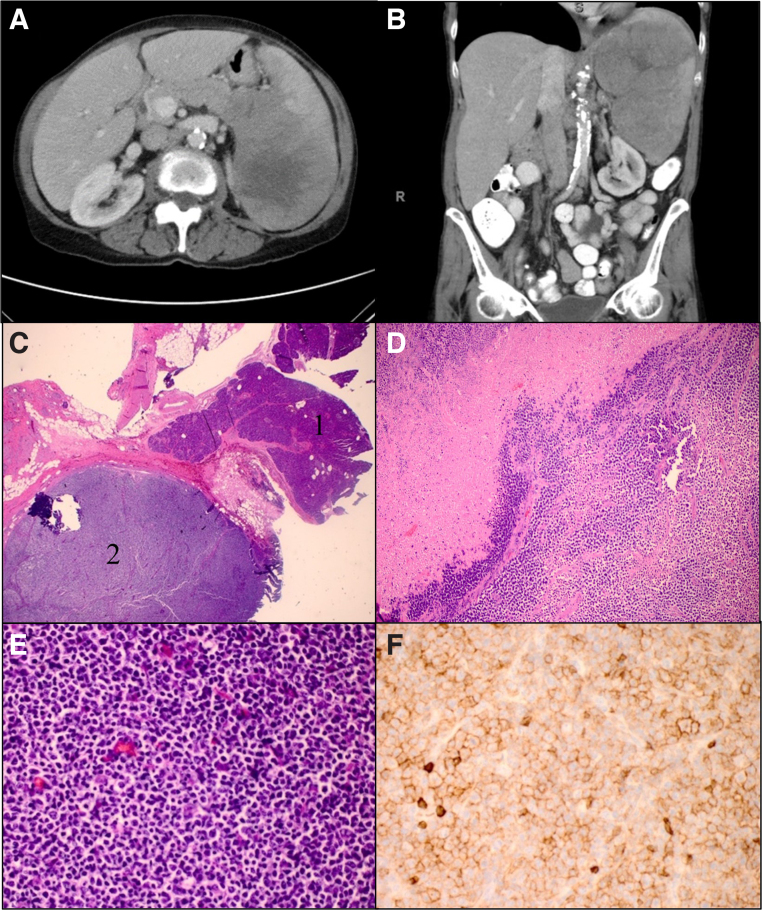
Axial **(A)** and coronal **(B)** computed tomography scan showing 12 × 12 × 18 cm mass at the tail of pancreas and splenic hilum. Histological sections of the lesion show that the lymphoma invades toward pancreas without infiltrating into pancreatic parenchyma **(C)** (hematoxylin and eosin × 20). Sections of the spleen reveal lymphoma cells infiltrating into splenic parenchyma with extensive necrosis **(D)** (hematoxylin and eosin × 40). Higher magnification shows a monomorphic population of large lymphocytes **(E)** (hematoxylin and eosin × 400). Immunohistochemical stain showing CD5^+^ expression by lymphocytes **(F)**.

The patient is a 64-year-old female who presented with left upper quadrant pain and abdominal distension. Computed tomography imaging (Fig. 1A, B) demonstrated a 12 × 12 × 18 cm heterogeneous mass with central necrosis that arose from the pancreatic tail and extended into the spleen. Suspicious lymphadenopathy was noted around the celiac axis, gastrohepatic ligament, and left pericardiophrenic angle. She underwent distal pancreatectomy and splenectomy. Intraoperatively, the mass appeared to originate from the pancreatic tail, abutted the stomach, invaded the diaphragm, and was surrounded by significant lymphadenopathy. Frozen section analysis demonstrated a high-grade malignancy consistent with lymphoma. Gross pathology showed a 19.5 × 12.4 × 8.2 cm tan-pink, yellow, fleshy, hemorrhagic, focally necrotic, and well-defined mass that protruded through a disrupted splenic capsule and grossly involved the pancreatic tail and surrounding soft tissue. The final pathological and cytological analysis showed diffuse large B cell lymphoma (Fig. 1C, F) arising from the spleen and involving the pancreatic tail, adjacent soft tissue, and lymph nodes. Morphologically, a diffuse pattern of atypical lymphoid infiltrate with sheets of large lymphocytes was present. These lymphocytes had large round nuclei, vesicular chromatin, small prominent nucleoli, and scant to moderate cytoplasm. Also noted were mitotic figures, apoptosis, and large areas of necrosis. Flow cytometry was positive for CD10 consistent with germinal center B cell (GCB) phenotype. Immunohistochemical staining was positive for CD5 and *BCL2* expression. The tumor did not express *c-MYC*. Fluorescence *in situ* hybridization (FISH) showed *BCL6/IGH* translocation, multiple copies of *BCL2*, and no copies of *c-MYC*.

Postoperative lactate dehydrogenase (LDH) was elevated at 443 U/L. R-CHOP (rituximab, cyclophosphamide, doxorubicin, vincristine, prednisone) therapy was initiated 2 months after surgery. Four months postresection, the patient had completed four of six chemotherapy cycles with good response.

## Discussion

We describe in this study the case of a patient with a lymphoma arising from the area around the pancreatic tail and spleen. Preoperative imaging and intraoperative findings suggested a primary pancreatic mass, but final pathological analysis demonstrated splenic lymphoma. Tissue biopsy was not pursued in this case but could be considered. It is important to note that fine-needle aspiration does not provide tissue architecture information and lacks diagnostic accuracy in the setting of lymphoma. Primary pancreatic lymphoma (PPL) is often, therefore, only diagnosed postoperatively^1–3^ and not commonly diagnosed in the tail of the pancreas.^4^ It is important to differentiate PPL from pancreatic adenocarcinoma because the treatment and prognosis vary significantly between these two entities.

As in this case, PPL usually presents with symptoms resulting from the mass effect of the tumor. Behrns et al. have proposed criteria for PPL, for example: (1) tumor predominantly located in the pancreas, (2) only peripancreatic lymph nodes involvement, (3) no hepatic/splenic involvement, and (4) normal white blood cell count.^5^ Treatment for PPL includes chemotherapy and surgical debulking, with better survival outcomes being associated with a combination of the two.^2,3,5^ Similar to PPL, primary splenic lymphoma (PSL) often presents with symptoms of mass effect rather. Similarly, it is often treated initially with surgery because the diagnosis is not clear preoperatively. In contrast to PPL, for which some authors advocate neoadjuvant chemotherapy and radiation, splenectomy is the first-line treatment for PSL, often with adjuvant chemotherapy and radiation.^6^

We present in this study a case of a large left upper quadrant mass, which preoperatively was concerning for PPL but was ultimately found to be consistent with PSL. The mass reported in this study is among the largest of those previously reported in the literature. Although diffuse large B cell lymphoma is curable, CD5 positivity is associated with more aggressive disease and a higher rate of recurrence with central nervous system relapse. Understanding the clinical and pathophysiological differences between these two manifestations of gastrointestinal lymphoma allows for more accurate diagnosis and treatment of these two rare entities.

## Abbreviations Used

FISH: fluorescence *in situ* hybridization
GCB: germinal center B cell
LDH: lactate dehydrogenase
PPL: primary pancreatic lymphoma
PSL: primary splenic lymphoma
R-CHOP: rituximab, cyclophosphamide, doxorubicin, vincristine, prednisone
